# Implementation and Evaluation of a Social Media-Based Communication Strategy to Enhance Employee Engagement: Experiences From a Children's Hospital, Pakistan

**DOI:** 10.3389/fpubh.2021.584179

**Published:** 2021-03-11

**Authors:** Muneera A. Rasheed, Alma Arshad Hookmani, Sana Waleed, H. Sundus Fatima, Soha Siddiqui, Muhammad Khurram, Babar S. Hasan

**Affiliations:** ^1^Centre for International Health, Department of Global Public Health and Primary Care, University of Bergen, Bergen, Norway; ^2^Department of Paediatrics and Child Health, Aga Khan University, Karachi, Pakistan; ^3^Smart City Lab NCAI, Computer and Information Systems Department, NED University of Engineering and Technology, Karachi, Pakistan

**Keywords:** online communication strategy, employee engagement, Facebook, paediatric, theory of change, patient-centered care, healthcare leadership

## Abstract

Social media can complement organizational communication strategy which is integral to employee engagement. However, successful case studies which can allow replication are limited. The objective of the study is to describe the design, implementation, and evaluation of a social media-based communication strategy in a tertiary care hospital in Pakistan. The leadership of the pediatric service line developed an intervention plan to engage the employees with the newly reframed vision to improve patient and family experience. An online communication platform—Facebook page—was created for all employees of the pediatric service line. The strategy to influence employees was based on Cialdini's six principles of persuasion. Implementation of the strategy between October 2017 and December 2019 was evaluated for reach, discussion themes, and outcomes using the framework by Murdough (2009). Quantitative indicators included total posts, mean comments, and reactions per post. Posts were qualitatively analyzed with an emergent approach for insights into the discussion. The analysis revealed a total of 9,085 posts, with mean reactions per post of 8.4, mean comments of 7.2, and active viewership by 90% members on average. In terms of post types, photos were the highest (4,779), while videos were the lowest (1,163). Qualitative analysis indicated 54% of the posts were of the theme “inspirational and thought provoking,” while the greatest engagement was generated on the theme “challenges and solution.” The authors conclude that the strategy was successfully implemented to maintain active membership, engage employees in meaningful conversations, and have them express intent to execute quality improvement projects.

## Introduction

Presently, communication is recognized as one of the most significant activities in organizations (1). A culture of effective communication enables the organizations to work toward such challenges by serving four main functions: control member behavior, foster motivation, provide for emotional expression, and provide information for decision-making (2). In addition to this, it further matures the organizational competences through “intense social and communicative processes” (3). Effective communication also allows groups and individuals to synchronize with each other on activities to achieve shared goals and assists them in problem-solving and decision-making and change management practices. In essence, it plays a fundamental role of building relationships among groups and individuals that the organizations prosper on. Additionally, to ensure performance management as well as the engagement levels of employees, effective communication has become imperative for the organization's success. Furthermore, research has also shifted its emphasis to the involvement of employees in decision-making and upward and downward flow of communication. Through effective use of communication skills, leaders can encourage employee engagement by proactively taking measures to ensure engagement concepts such as job satisfaction, motivation, and organizational citizenship behavior. Moreover, faith in a leader, support from the leader, and forming a human-centric environment are reflected as factors of psychological safety that also leads to employee engagement, as proposed by Kahn (4).

Evidence suggests that managerial communication should be repeated several times using a variety of different methods in order to be truly effective (5). The numerous channels of communication used for internal communication include face-to-face meetings, phone calls and conference calls, e-mail, instant messaging, and videoconferencing as well as social media. In comparison, communication has transformed more the rise of social networking (6) leading to considerable changes and prospects for both business and social communication. Social media like Facebook, LinkedIn, Twitter, and blogs are being used in corporate practices for communication and engagement with both employees and customers. Kolzow (7) established that social media has empowered leaders to not just improve their communication skills but also connect with people both inside and outside at a much deeper level. The use of social media is considered as part of transformative culture (8), providing various benefits when it comes to organizational communication. Embracing social media at the workplace causes levels of employee engagement, teamwork, and information management to rise. It also enables gathering and sharing of data on various social media platforms, which may be used as an internal tool to gather, share, and maintain data by the organization (9). Likewise, it also allows the leaders to keep an update with the demands of employees and maintain innovation (10). Through the prevalence of constant advancements, leaders have to adapt their approach as well as their thinking to connect with employees online. Managing an effective social media presence requires time and intensive effort. Nonetheless, by embracing this opportunity and investing both time and energy to connect with employees in a transparent and timely manner can shape the company values and how it is perceived in very powerful ways (11).

Emerging evidence indicates that a change in organizational communication can lead to engaged employees. The healthcare organizations are no different when it comes to achieving their own targets in the form of positive patient outcomes by shifting their focus on employee engagement (12). Additionally, operations in healthcare settings possess a dire need for empathy-based and trustworthy relationships. Both of these aspects encourage people to work and play together online as well as offline (13, 14). Promoting group relationship, teamwork, and harmonization is an essential element to ongoing quality improvement practices (15). An online mechanism along with physical communication and interaction can serve to mobilize the participants. In addition to this, some studies brought light to the significance of encouraging healthcare staff to voice their concerns. Maxfield et al. (16) in the Silent Kills study found that even when encountered with setbacks that threatened patient care, about 90% of health professionals still remained silent. Even physicians would “swallow” their discomfort in similar situations [(17), p. 304]. Moreover, since the nature of work of healthcare workers especially nurses is very demanding and emotionally exhausting, they tend to feel burnout (18). Hence, they should be given opportunities to show their concerns as direct voice is negatively related to burnout (ibid.). The current study uses a communication platform to fulfill the aim of enhancing an organization's functioning by removing limitations related to vocalizing concerns. A unique characteristic of this strategy is that the workers can share their grievances and worries anytime they want by posting on the Facebook group.

Though good communication in terms of interacting face to face is rated to be extremely crucial in clinical teamwork settings (19, 20), however, there has been limited research on communication through social platforms. In fact, many organizations and healthcare institutions have avoided to shift along the lines of cultural transformation by adapting public social communication, due to the risks and ethical issues, reputation, privacy, productivity, training, and education associated with it (21). Latest evidence about the effective use of social media-based communication strategies may help to overcome such criticism especially when work is now largely virtual in the face of the pandemic (22). Successful models will be needed in all organizations but specifically healthcare which is under immense pressure at the verge of breaking down due to the workers facing physical and psychological pressures (23–25). The current study aims to describe experiences from one such model implemented in a healthcare organization. The leadership of the Children Hospital service line had reframed its vision. This implied including patient experience of care as an integral part to optimum services. It was also identified that the pathways to patient experience improved employee experience which meant a huge cultural shift for a largely physician-centric organization. Hence, a social media strategy was devised and implemented to facilitate the transformation. The objective of the study is to describe the design, implementation, and evaluation of the strategy.

## Methodology

### Setting and Sample

This study was implemented in the pediatric service line at Aga Khan University Hospital (AKUH), established in 1985. It is a Joint Commission International certified and categorized as a private, not-for-profit. The service line caters to children from birth to 18 years of age with in- and out-patient services and consists of diverse employee cadres providing care to children and adolescents with different health problems. Since all employees of the service line were eligible, a population-based sampling was used. The study sample consisted of about 600 employees working for the service line, out of which 61% were females and 39% males. They were from the following professions: nursing (60%), faculty (11%), trainee physicians (14%), and admin staff (13%), and the rest were allied professionals and research staff. The study was implemented as part of a quality improvement project for improved patient experience of care by enhancing employee experience of care. The study was approved as a quality improvement project by the Aga Khan University Ethics Review Committee.

### Role of the Communication Strategy

The communication strategy was designed to augment the delivery of intervention of employee engagement based on the framework (26) as described in Table 1. The framework speaks about enablers that enhance employee engagement through leadership, engaging managers, employee voice, and integrity, which are also known as the “four enablers.”

**Table 1:** Role of communication to enable employee engagement.

**Enabler**	**Role of the communication strategy**	**Application on the Facebook page**
Leadership	To connect with employees, share the vision of the organization, and answer employee's questions so they could relate their work with the purpose.	The purpose and vision will be communicated in the “About” section of the “Batti Factor” group and also on the posts by leadership.
Engaging managers	To send a silent message to the employees that “Your organization cares about you,” which lets the employees feel connected, naturally leading to improved motivation and performance.	The leaders will make an effort to engage managers through modeling behaviors of consistent posts and responsive comments. The posts will revolve around topics of supervision, coaching, encouraging staff, and mentorship to create new values of effective management.
Employee voice	To hear employee challenges from bottom up and facilitate resolution in a prompt fashion. To allow employees to share what they felt was important to them.	Employees will be encouraged to voice their concerns through commenting on or making posts on the group regarding their challenges.
Integrity	To showcase alignment between claims from the leadership and their actions.	Leadership conversations will indicate value for patient experience and employers who provide optimal care will be duly recognized.

### Gap Analysis

The leadership analyzed the existing communication system and discovered certain gaps that could be barriers in creating awareness of the vision and engagement among employees. The analysis further revealed that the existing communication strategy lacked a proper flow of both formal and informal information. The formal communication existed both horizontally among the departments and vertically that is from the top to the lowest level of the hierarchy. However, the vertical communication only existed up till a certain level of hierarchy, with the lower level employees being unaware of most of the important information. The informal communication was close to none leading to two main issues: approachability and emotional engagement. The employees felt that the leadership was unapproachable, and furthermore, they did not know each other apart from their team members. They sensed an absence of outlet and no value of their opinions, which was the reason behind their disengagement. In addition to a dearth of a consistent feedback system, the policies were also not communicated properly, causing unawareness of a clear vision and, hence, no implementation. Hence, after a quick needs' assessment, communication strategy was redesigned, keeping the employee engagement at the center. Blatant to the technological advancement in this era, this only seemed achievable through the usage of social media. The team chose Facebook as it could incorporate both formal and informal communication for the employees as well as leaders.

### Design of the Strategy

**Figure 1:**
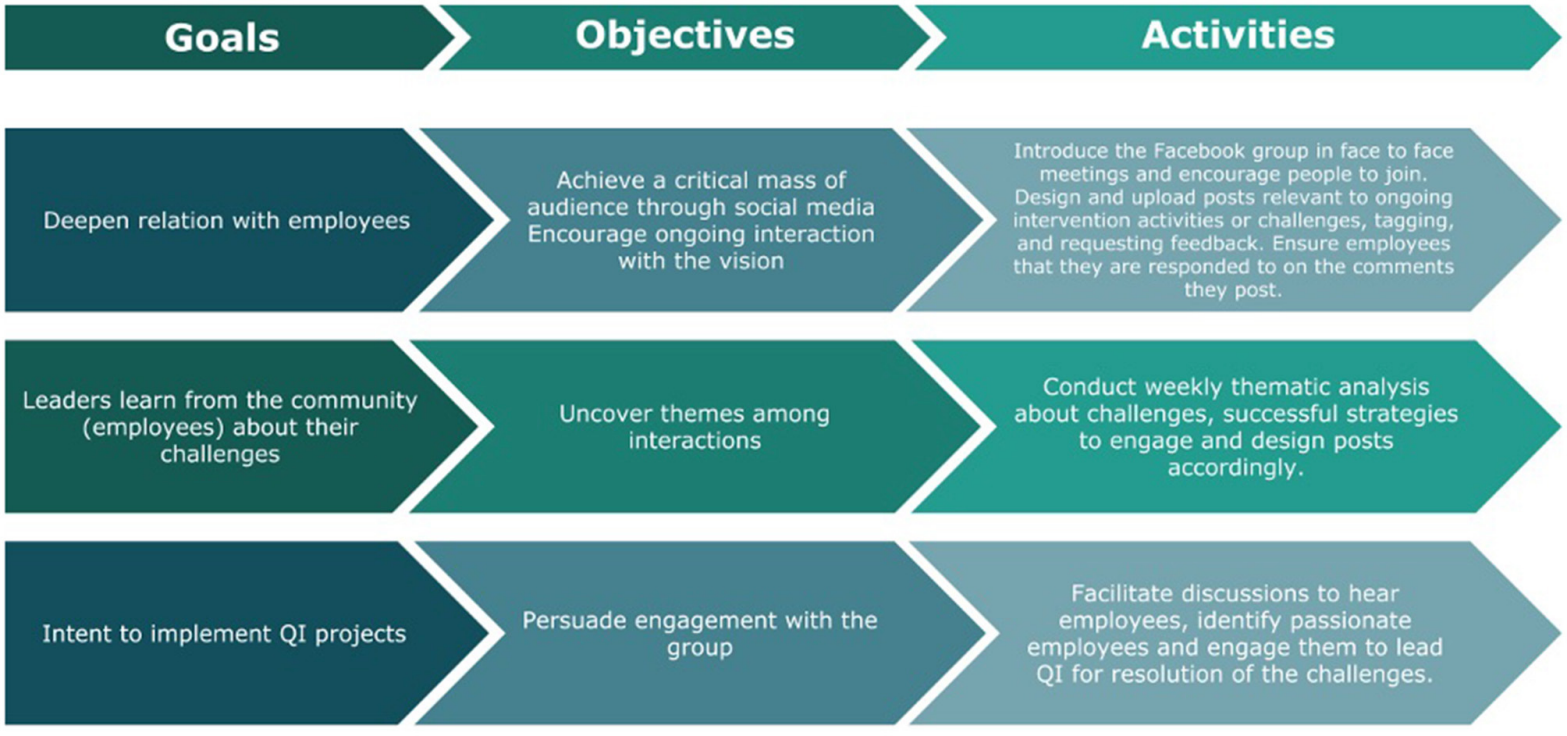
Goals for implementing a social media based communication strategy. Adopted from framework by Murdough (27).

The team created a Facebook group—“The *Batti* Factor,” which means inner light or spark which rages into fire of passion to deal with the grand challenges at the workplace and beyond. The page was meant to include all the employees of the service line. The implementation followed three broad goals with specific objectives and activities (Figure 1). The first and foremost goal was to develop or maintain deep relationships with the employees achieved through bringing a critical mass of employees to the group while encouraging interaction with the vision. Connected to it was the next goal to uncover themes and underlying issues for the leaders to become aware of employee challenges through acknowledging and listening. Once heard, the last set goal was to engage employees to express willingness to implement QI (quality improvement) projects for the challenges identified led by the employees themselves. The objectives were facilitated through ongoing discussions between the employees and the leadership.

In order to influence the employees, the vision was communicated, through commencing approaches based on the six principles of persuasion (28).

- “Reciprocity”: The leadership positions were mentored so that they are prepared to guide the conversations and engage the employees. When they appreciate and show concern, that is when the employees feel they must reciprocate the same positive attitude.
- “Commitment and consistency”: The principle of consistency is based on the power of active, public, and voluntary commitments, which results in people actually sticking to their word. When engaging with online discussions, the members would commit to support the QI.
- “Social proof”: When people are uncertain, they will look at others to make their own decisions. When employees presented their QI projects or participated in an activity, it was ensured to be communicated.
- “Liking”: A tactic was devised that in responding to comments, people similar in nature or personality to the commenter will respond. The employees feel more connected and can relate to people they like.
- “Authority”: The leadership/supervisory positions were mentored to post and respond to comments of the employees in an empathic assertive style. This included the Service Line Chief (SLC), the managerial positions, and others.
- “Scarcity”: The leadership introduced quality projects and communicated to the employees that such initiatives have limited seats available, which seemed to be exclusive to the employees, motivating them to participate more.

### Implementation Procedures

For the first 6 months, the task of designing the posts was assigned to a core working team which consisted of a communication consultant (for the first 3 months) and members belonging to leadership roles. The core members were responsible to engage the employees with the posts. There were standard operating procedures (SOPs) for every core member to post daily. Each core team member posted with the intent to engage staff from their respective group, using the principles defined above. Moreover, the leadership consultant also posted occasionally, followed by the core working group.

The implementation of strategy in the first 3 months (October to December 2017) led to a large number of posts which was overwhelming for the team to analyze them thematically. Hence, a proper schedule of posts was formed which stated what to post on a specific day and what is the purpose of it. On Monday, the group had posts of “summer day” which consisted of positive points of the past week as a reminder, while Tuesday was reserved for “voting for interesting topics” and discussions taking place throughout the week on the selected ones. “Gratitude” posts were shared on Wednesdays to spread positivity, whereas discussions on “grievances” were done on Thursdays and “appreciation” posts for individual members or groups were presented on Fridays.

In addition to this, the following ground rules were also created for the core working group:

1. Every member has to respond to a question addressed in the group.
2. One new idea/member/week: This idea will revolve around the transformative process. How can we make it better? How can we learn from data to make the process effective? How can we increase engagement? Any ideas regarding data analysis? How can we increase the success of QI projects?
3. Every member will have to put a post every alternate day on the group. The post has to be thoughtful. Discussion should be provoking and should revolve around solutions to pain points.

As part of the monitoring strategy, the team kept a record of all the posts. In the first few months, data was analyzed each week followed by monthly analysis. The following quantitative indicators were selected as provided by Facebook insights and sociograph:

- Percentage of active members (members who have viewed group content) in a given time period
- Number of total posts uploaded
- Mean comments (the average amount of comments on a single post)
- Mean reactions (the average amount of reactions on a single post)
- Mean ratings (calculated as 3^*^comments + 2^*^likes) per post

Analysis also included the top contributors and most viewed posts. Another objective was to develop an understanding and become aware of the posts that interest employees the most and how that could be utilized in increasing their engagement levels. The posts were recorded in an Excel file by a research associate (an organizational psychologist) with the title, person who posted it, date, type (status, photo, video, or link), number of likes and comments, and thematic analysis of the comments and were coded according to their themes. The themes were coded based on the central idea of the posts; e.g., if a post was meant to inspire and motivate or bring/encourage positivity, then it was coded as “motivational and inspirational” or “positive change.” The analysis was supervised by a core group member who was assigned the responsibility (last author). The analysis was discussed among the team and relevant decisions were taken to design the strategy for the next week.

### The Evaluation Framework

Implementation of the communication strategy was evaluated using a framework by Murdough (27) customized for the goal of employee engagement. The original framework was presented for social media programs targeting marketing. The metrics were around three pillars framed to navigate the complexity of the social media data (Table 2). “Reach” assesses the extent to which the team was able to spread their message of the respective strategy. This was measured through the base-level indicators such as the number of members and the posts uploaded in the group along with the profile examination of the added members. “Discussion themes” caters to the ways employees are being engaged in the group and how it is being measured. This is measured by observing ratings, comments, and topics/themes per post. On the other hand, qualitatively, thematic analysis, leadership roles, and discussed topics on the group are noted. “Outcomes” of the employee engagement was evaluated by estimating the number of employees expressing intent to implement QI projects and how many of these projects are linked to the reframed vision.

**Table 2:** The evaluation framework.

**Pillar**	**Quantitative indicators**	**Qualitative indicators**
Reach	# of members # of posts uploaded	Profile of members
Discussion themes	Topics/themes per post Mean ratings of posts Mean comments of posts Mean reactions of posts	Topics/leadership roles Thematic analyses of the posts
Outcomes	# of employees who expressed intent to implement QI projects	QI projects linked to vision of the organization

*QI, quality improvement*.

An engagement survey was designed to evaluate the ongoing strategy. The survey explored whether the employees viewed the engagement initiative to be useful by incorporating questions such as whether they are aware of the strategies for the respective incentive, which one was the most effective, whether they themselves were active, and the reasons for engaging on the Facebook page. The responses from the questions based on the Likert-type scales were summarized and analyzed using descriptive statistics. A few open-ended questions were also asked in the survey which included their preferences on the type of posts they would like to see, the changes or challenges they have observed during or after this initiative, and any suggestions. This was asked mainly to develop comparatively new and better strategies.

### Data Management and Analysis

Quantitative indicators were collated using the *sociograph* software (29). The indicators remained the same as used in the monitoring strategy. For qualitative analysis, the data about each post was gathered on a daily basis in terms of the posts, reactions, views, and summary of comments each post received on an Excel sheet. These were thematically analyzed by an associate and reviewed by the senior author for agreement using an inductive approach (30). Analysis is presented as frequency and percentages or mean as applicable. The evaluation data was interpreted in accordance to themes and time frames. It was essential to opt for these approaches together because the authors felt the engagement should not be evaluated just by analyzing the themes that emerged throughout but also by being aware of how the levels of engagement changed over time and the underlying factors behind it. The analysis examined all the posts and sorted them according to their purpose (theme) and then presented as a time frame evaluation, emphasizing on the engagement and most recurring theme and the possible factors that may have influenced employee engagement.

## Results

### Reach

**Figure 2:**
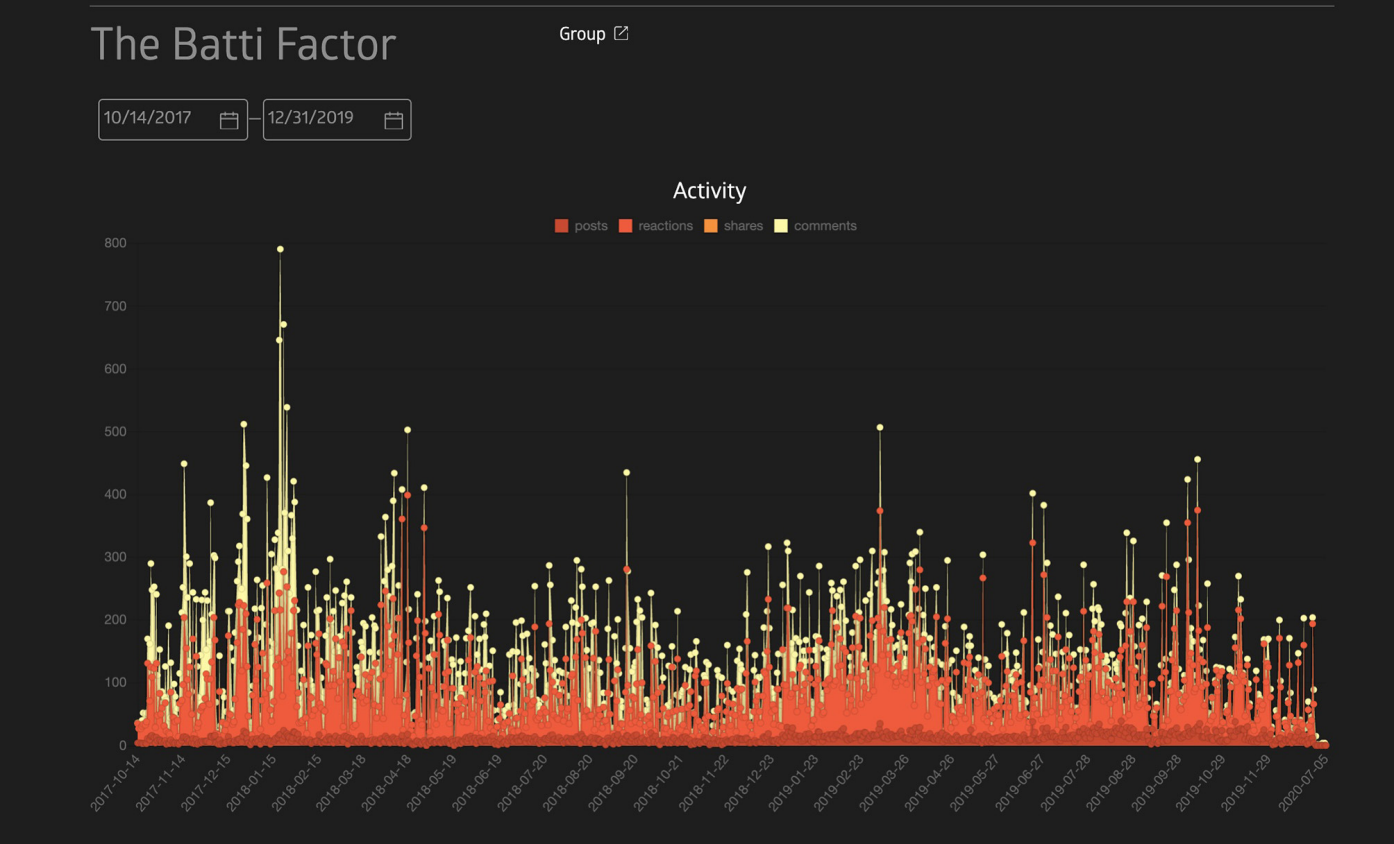
Group activity: October 17 to December 19. Source: Sociograph.

In the first 3 months of the intervention, about 130 members had joined the Facebook group with ages of 18–46 and above. There was a gradual increase in members which was noted yearly till 2019 when there were altogether 625 members by December 2019. The data suggested that the number of active members has been mostly constant throughout (90%). An analysis of posts between October 2017 and December 2019 indicated a total of 9,085 posts (Figure 2).

### Discussion Themes

Of the 9,085 posts, total reactions of 65,930 and total comments of 63,446 were recorded with mean reactions per post of 7.3 and mean comments of 6.9, respectively (Table 3). In terms of post types, photos were the highest (52.9%), while videos were the lowest (12.8%). The thematic analysis indicated six broad themes: challenges and solutions, informative, inspirational and thought provoking, acknowledgment and appreciation, recent updates, and compassion (Table 4). Each one having a different dimension made a major contribution in terms of aggregating engagement and building a robust network. Collectively, there were more than half posts for “inspirational and thought provoking” (54.5%), and the rest of the thematic posts were below 20%. Even though the posts for “challenges and solutions” were the lowest (0.3%), the employee engagement as indicated by mean ratings (111.2) and mean comments (30.6) was the highest.

*Challenges and solutions:* The theme “challenges and solutions” entailed all the posts that were related to challenges not only based on patient experience or organizational level but also catered to the limitations faced by the employees as an individual.
*Informative*: This theme served the purpose of educating regarding numerous topics that connected the employees to the organization and its patients at a whole new level. This also involved providing them with newsletters, insights from a recent article, useful tips that can be applied professionally, and other valuable information.
*Inspirational and thought provoking*: An important part of communication is to converse with the employees in a manner that inspires them and encourages them to think “out of the box.” The posts for this respective theme were selected with the aim that employees mold themselves in every aspect and become a better version. The content of this theme included how to act when dealing with people, inspirational stories, never give up or lose hope, emotional mastery, motivational quotes, etc.
*Acknowledgment and appreciation*: “Acknowledgment and appreciation” served the purpose of appreciating the employees for their achievements and hard work. In order to appreciate them at an individual level, an initiative called “Person of the Week” was introduced in the first half of 2018. A total of 82 posts appeared from this scheme. This was different from the other posts as the person was selected on the basis of nomination. Anyone in the group could suggest a nomination with the page administration team under a criterion shared on the group which included not just being good at work but also showing compassionate behaviors. The “Person of the Week” was announced every Friday with a picture and a paragraph written about the person's strengths. This post in particular was the most anticipated and received the most likes and views (Table 5).
5. *Recent updates*: This was originated to keep the members informed and up-to-date regarding the happenings of organizations. The posts were mostly regarding organizations' workshops, sessions, meetings, or events taking place. It was believed that this initiative further increased employees' trust in the organization.
6. *Compassion*: An essential requirement for working in a healthcare organization was to be compassionate to others. Hence, numerous posts regarding soft skills were presented to develop the feeling of compassion in employees. Another reason for this theme was that sometimes participants also face indifferent encounters from their mentors, but they are unable to confess about it. Therefore, this theme provides a feeling of comfort that the employees are being heard and understood.

**Table 3:** Data trends of the Facebook page over the study period.

	**October–December 2017**	**January–June 2018**	**July–December 2018**	**January–December 2019**	**Total**
Total *N* (%) of active members	69	93	91	90	–
*N* (%) posts	580 (6.3)	1,481 (16.2)	1,529 (16.8)	5,501 (60.5)	9,085
Total reactions	5,752	17,035	11,080	32,063	65,930
Mean reactions	9.9	11.5	7.2	5.8	7.3
Range	0–87	0–110	0–153	0–134	0–153
Total comments	7,080	9,049 + 4,824	8,878	12,662	63,446
Mean comments	12.2	9.4	5.8	2.3	6.9
Range	0–151	0–228	0–94	0–124	0–228
Mean ratings	52.3	50.9	29.7	17.6	28.5
Range	0–477	0–728	0–588	0–481	0–728
**POST TYPE**					
Photos (%)	266 (45.8)	664 (45.0)	711 (46.5)	3,165 (57.5)	4,806 (52.9)
Videos (%)	105 (18.3)	274 (18.6)	213 (13.9)	575 (10.5)	1,167 (12.8)
Links (%)	42 (7.2)	198 (13.4)	221 (14.3)	917 (16.6)	1,378 (15.1)
Statuses (%)	167 (28.7)	339 (22.9)	384 (25.1)	844 (16.1)	1,734 (19.1)
POST THEME^*^					
Acknowledgment and appreciation	10 (3.5)	232 (12.1)	157 (11.4)	547 (12.7)	946 (11.9)
Challenges and solutions	10 (3.5)	12 (0.6)	0 (0)	0 (0)	22 (0.3)
Compassion	5 (1.7)	78 (4.1)	50 (3.6)	200 (4.6)	333 (4.2)
Informative	11 (3.8)	318 (16.5)	233 (16.9)	884 (20.5)	1,446 (18.3)
Inspirational and thought provoking	218 (75.7)	1,013 (52.7)	838 (60.7)	2,244 (51.9)	4,313 (54.5)
Recent updates	34 (11.8)	269 (14)	102 (7.4)	446 (10.3)	851 (10.8)
Total	288 (67)	1,922 (97)	1,380 (97)	4,321 (83)	7,911 (88)

* *Thematic analysis was completed for N = 7,911 (88%) posts*.

**Table 4:** Summary of posts by themes over the study period.

**Themes**	**Definition**	**Examples of posts [Post story, date posted]**	**Engagement indicators**		
			**Ratings range and mean**	**Reactions range and Mean**	**Comments range and mean**
Challenges and solutions	The challenges faced by employees on individual and organizational level, with their solutions.	Tell me only one thing that hinders your FCPS passing? (Status, November 2017)	6–728 111.2	1–62 9.6	0–228 30.6
Acknowledgment and appreciation	Employees being appreciated for their hard work and achievements. This also included the “Person of the Week” posts.	Appreciated for 100% Attendance in NICU (Status and photo, September 2018).	0–588 56	0–153 15.1	0–118 8.7
Recent updates	Keeping the employees updated with the organizations' activities. This includes meetings, events, trainings, sessions, etc.	Central line care workshop in NICU (Photo, February 2018).	0–392 42.5	0–119 11.2	0–94 6.7
Compassion	The posts focused on developing soft skills in the employees to have harmonious interactions with each other.	Know that you and your colleagues are in the same boat. Be kind to each other. Learn from each other's strengths (Photo, January 2018).	0–300 27.4	0–110 7.5	0–71 4.1
Inspirational and thought provoking	The posts helped employees to think “out of the box” and shape themselves in every aspect.	What do you people think about work life balance? (Photo, April 2019)	0–512 25.8	0–110 5.1	0–152 4.5
Informative	Provide employees with information regarding their field, organization, and patient care.	Research paper about the positive relationship between employee motivation and performance (Status and link, November 2018).	0–296 19.3	0–70 4.8	0–71 3.2

**Table 5:** Employee feedback about being selected “Person of the Week”.

**Quote (employee cadre)**
“*I would like to share my experience when I was awarded with the ‘Person of the Week. It is a huge honor for me to see my name at this label. I was extremely happy with this title and felt good knowing that my qualities are recognized that I myself have never concentrated on. After this I was motivated to work more. According to my opinion, if you appreciate an employee's hard work and determination this leads to a lot of positive change in him/ her and he/ she becomes more sincere to his/ her work*.” (ID 23, October 2018)
“*It was quite surprising for me. I never expected that it could come my way too. Since there were other more experienced and skilled professionals already in line, I never thought of having it, at least for myself. When I was nominated for the title, I felt very excited and humble at the same time. The very feeling of being in that list of valued employees of the hospital was priceless. Above all, without my mentor I would not have bagged this title. Her personal efforts were instrumental*.” (ID 41, February 2019)
“*I was so pleased and motivated to know that, since it was the* first *time; like an appreciation coming from leadership. It should continue. Also, I would like to highlight that just after my appreciation, I saw another member who was selected for the ‘Person of the Week’, which was so unjust and biased. It seemed like it was just because of jealousy and if I have received* one *so should he/ she. Moreover, I also got to know that the person who got the appreciation had forced his/ her faculty to write a post for him/ her, which according to me, decreased the value of appreciation*.” (ID 15, August 2018)
“*It felt great, satisfying and encouraging to be highlighted amongst the group. It gives a sense of achievement and acknowledgment and also gives the reflection that yes, our superiors are acknowledging our hard work and efforts and it is indeed a proud moment for not only myself but for my family as well*.” (ID 1, January 2018)
“*It felt great to be noticed and appreciated. It was a very sweet gesture and it made us want to work harder! As far as my personal experience is concerned I felt accepted, satisfied and that also increased my motivation and love for my work even more*.” (ID 40, January 2019)
“*I had an amazing experience working under my mentor and it was an honor to be selected as the ‘Person of the Week’ because it always gave me an immense level of motivation*.” (ID 46, March 2019)
“*It is an amazing idea and gives motivation to continue working at best. I feel that someone has recognized the work I do. Nurses also feel proud and those who didn't become ‘Person of the Week’ get motivated*.” (ID 24, November 2018)
“*I felt special and appreciated. I was not expecting it. I also felt gratitude toward my mentor and the leadership of the pediatrics department*.” (ID 11, June 2018)
“*It was a nice feeling. I read every word of it multiple times and felt valued. It's good to get this kind of affirmation once in a while*.” (ID 30, December 2018)

An interesting thing to note was that the four enablers (MacLeod and Clarke, 2019) were also submerged in these comprehensive themes, with instances of overlap. The enabler “leadership” comprised posts about significance of patient experience and tied it with larger human experience values like compassion; “employee voice” was mainly concerned with “challenges and solutions” as it raised employee concerns; however, employees also made posts around other themes. Lastly, the enabler “engaging managers” included posts around engaging employees through supervision and mentorship which were covered under the theme “recent updates” and “informative” while also appreciating employees through “acknowledgment and appreciation” posts.

### Outcome: Expression of Intent to Implement QI Projects

Employees expressed intent to engage with QI projects and regularly updated on the page about their plans and execution status. The content of QI projects came from facilitated discussions based on employees' concerns, challenges, and passions. The said initiative began through a post uploaded by the SLC, which stated:

“*One problem*, one *solution challenge. This challenge entails identifying 1–2 of your colleagues in SL-4 who should identify* one *problem that deals with patient care and* one *high quality solution to solve it. I identify that patients are not greeted when they come to the ward?*

*Solution: for every admission the respective team leader (nursing) and senior resident of that team will go and greet them and tell them what to expect. I challenge SI, MR, ZM, JJ, AI, RH and SG*.”

(Status, SLC, October, 2017)

Many participants actively responded to the above post, of which a few responses were as follows:

“*The most painful challenge is seeing that the patient is not treated with respect and dignity at times*.

*Solution: Teach staff to treat people with respect by modeling and treating the staff well. Ensure a clear mentorship strategy for all the staff dealing directly with patients*.”

(Comment on Status, Faculty, October 2017)

“*Respect for Juniors. Solution: Before the rotation, discuss their expectations. Treat them as colleagues, train them, feel accountable to them. At the end of the rotation, get face to face feedback, ask them what they would like to be changed? Juniors love to know that their opinion matters, they feel empowered and that motivates them. Make their experience so enriching that they want to join SL4!!*”

(Comment on Status, Faculty, October 2017)

“*I feel bad when a child looks at me and starts crying although I didn't touch him … so I see there is a lack of rapport and trust between the nurse and patient. The concept of children is that nurses are there only to hurt for e.g., insert cannula or administer medication or do suctioning which gives them a painful experience and thus creates a negative image of a nurse. Solution: A specific time should be allotted for play therapy, we have a small play area and I think we should utilize it well. Assigned nurses may take their patient's to play area and spend a little time with them, this will help in gaining their trust and build a good rapport*.”

(Comment on Status, Nursing staff, October 2017)

“*Challenge for patient escort from admission office to inpatient areas. Solution: there is some designated SL04 staff, he/ she greets, escorts new patents from admission office to wards and teach basic facilities which are provided by hospital during patient stay (not clinical), e.g., water cooler, attendant's food, visiting time, feeder sterilizer, prayer area and play area*.”

(Comment on Status, Service coordinator, October 2017)

As part of the initiative, participants frequently provided updates on their or others' RPMs by uploading status, video messages, and even photos:

“*The most awaited and the thrilling update by Dr. NA on his RPM..!!*”

(Video message, Faculty, January 2018)

“*Dr. PC RPM update and gantt chart*

*Outcome: To give absolute sense of certainty to the parents of children with epilepsy by creating awareness about the key steps at the time of active seizure*”

(Photo and video, Trainee physician, January 2018)

“*Dr. IN is telling us about his RPM focusing on residents and fellows involvement in research*.”

(Photo and video, Faculty, January 2018)

“Rocking OR [Operating Room] to Cardiac to Pediatric ICU [Intensive Care Unit] ha*ndover RPM by Dr. MM with QA SA and TF. Great work and good luck!!*”

(Photo and video, Faculty, February 2018)

Moreover, a few participants even shared the impact of RPMs and appreciated the members who contributed toward planning and executing it:

“*The impact of Dr. AF's RPM was not confined to Faculty, Residents and students*.

*Our Nurses felt equally empowered. Here are nurses sharing their perspective*.”

(Video, Communication consultant, January 2018)

“*Posting after a while. I really want to give a shout out to SS for leading the meeting with emergency medicine for our handoff RPM. I have worked with SS as her supervising fellow when she was a resident, as a faculty supervisor while she was in research and now as her fellow colleague. SS has evolved as a teacher and as a physician and is showing increasing skill and maturity, providing innovative solutions to complex problems, all with grace and compassion. She has always gone out of her way to help everyone (yours truly included). Thank you SS. You are a leader.”*

(Status, Faculty, February 2018)

Feedback was also taken in form of an uploaded status on the Facebook group:

“*Are all team leaders enjoying the RPMs activity? Team members please give your feedback about your team leaders and if you are facing any challenges please comment. Below is the list of RPMs with their Team Leaders. If I forgot anyone..please add them*.”

(Status + photo, Faculty, January 2018)

### Principles of Influence

This section expands upon the posts that were designed following the Cialdini's principles of influence and used as a means of engagement. Even though every post was intended to be based around a single predominant principle but the discussion on the themes entailed other principles for example, real challenges were highlighted and responded to by the core group members (reciprocity), taken notice by the higher leadership (authority), and engagement led by the immediate supervisor (liking).

1. “Reciprocity” was observed to be present in most of the posts to encourage the employees to connect, discuss real issues and challenges, and provide an opportunity to hear and empathize with them. One example in which a nurse manager posted regarding reducing nursing documentation:

“*CNI (Clinical Nurse Instructor) NN of PICU (Pediatric Intensive Care Unit) is leading the RPM [QI] of reducing nursing documentation in children's hospital along with Farheen from outpatient. So, I request all the staff who want to join them: please come ON. A great opportunity to bring about the change*.”

(Status, Nursing manager, January 2018)

Moreover, posts based on this theme were also meant to appreciate employees contributing in a positive manner to the respective initiative and encourage others to improve their performances. For instance, communication consultant Khan appreciated the five contributors on the group and posted a video along with a status which stated:

“*A loud shout out to our top 5 contributors on the group! You guys are simply awesome and your engagement on the group is highly appreciated!!*

And for the rest of the crowd! Get your A Game ON! This is happening for YOU!

*High Fives!!*”

(Video, communication consultant, January 2018)

2. The notion of commitment and consistency was visible in posts where employees shared a challenge that upset them or were engaged during discussions to commit to lead QI projects or support resolutions by the respective supervisors and leaders. Two examples of posts that depict this principle are mentioned below:

“*We need to equip ourselves with skills which help our girls thwart malicious intent but more importantly as medical practitioners help the zainabs who survive, recover and thriv*e.”

(Status, Physician, January 2018)

“*Finally!!! Launched the resident mentoring RPM (Rapid Planning Method)*. First *session of the principles of research methodology course underway by …. The objective of this RPM is that our residents produce best quality research which is publishable in high impact journals and not merely done as a requirement for CPSP. The course itself will be conducted by faculty from the department of pediatrics who have extensive experience in pediatrics and public health research and will be a mix of didactic sessions and hands on exercises on epidemiology and data analysis. The course is open for all residents and fellows but mandatory for year* one *residents. Outcome is to have a synopsis developed and submitted by the end of year one*. Second *part of RPM will be* one *to* one *pairing of each resident with a research faculty for mentoring along with their supervisors*.”

(Status + photo, Physician, July 2018)

3. Social proof was brought in through the intervention activities that were shared on the page or giving examples of other members to encourage others for participation.

“*Don't be ashamed of your story … it will inspire others. Who has some real stories to share? Let's get the word out!*”

(Status + photo, Communication consultant, December 2017)

“*For all those who wanted to know the power of this page—Here is just* one *example of what YOU can do! Issues that never were talked about and addressed in years are now on the table and actions are being planned to resolve them. Now is the time to speak up and take accountability of your actions and get in the movement of change because it's YOU who will bring it and it's YOU who is trusted by the leadership for this transformation*.”

(Status + video, Communication consultant, January 2018)

“*Here's how SS spreads the Batti in the RPM presentations on Tuesday, November 14, 2017. His energy was on, his physiology was game on, and his batti was completely charged up and everyone could feel it! This is what we are looking for in RPM. If you know your purpose, if you know why you're doing it and the importance of it for YOU. There's no stopping from there man! High* Five *SD*”

(Status + video, Communication consultant, November 2017)

4. The principle of “liking” arose in posts that received elevated responses through likes or comments. This was mostly observed where supervisors voluntarily or after encouragement responded to posts uploaded by the staff, since appreciation from the supervisors mattered. Examples of these posts included where appreciation was shown in numerous ways such as announcing the “Person of the Week.”

“*Congratulations Mr. WD-Person of the Week*”

(Status + photo, Research team member, February 2019)

“*I am feeling so honored to share that in yesterday's nursing Grand Round, all the appreciation was shown for 2017. And Children's Hospital had the highest number of appreciations throughout the year. THANK YOU ALL FOR MAKING US PROUD*!”

(Status, Nursing manager, April, 2018)

5. Authority was incorporated through members in leadership roles, making special efforts to share or respond to different posts in the group.

“*Who wants to criticize and who wants to fight? Let us all pledge to know the lows of failure and the high of success. Let us all pledge to solve rather than complain. Who is with me on this one? Say yes or mark like*.”

(Status + photo, SLC, November 2017)

“*The TOC workshop? Yes but much more: This is a demonstration of the dedication of the SL4 leadership toward the patients. You've never seen this before in AKUH—the Academic Chair, the SL Chief, the Business Manager, the Nurse Manager and senior doctors meticulously working with the SL team to ensure the execution & delivery of the RPM's at a superior level. This quality of commitment & energy is what it takes to give a sense of certainty to the patient—it's not easy to change habits, mindsets and actions but it can be done. This is how it's done and that's why we're able to achieve results in months vs. the traditional years! Over the last 6 months the Core Team has consistently led by example; making the difficult decisions, doing the hard work and putting the team first. That's how the RPM teams were created and that's how the RPM teams have worked for months also. No egos & no ranks—all* one *team! When we celebrate our victory, people will only see the end result, they will not see the days, weeks, and months of hard work we all put into this, but we will know—that's why this is a labor of love—Batti!!*”

(Status + video, Leadership consultant, April 2018)

6. Lastly, engagement was prioritized by implementing the principle of scarcity which involved having posts on opportunities of participation in different trainings, events, or sessions.

“*Anyone interested in the responsive play training pls inbox me or comment here. An inspirational training to learn how-to engage kids and maximize their potentials, be all set to handle your kids nephew's niece grandkids. Open to everyone in SL4, nurses, physicians, SLC, chair, CEO too*.”

(Status, Faculty, November 2017)

“*This is a once in a lifetime opportunity to learn, grow, and contribute to a rare transformation event. When you feel that sense of certainty and find yourself ready to make a decision to be part of this team, show up to the meeting on Friday 17th November at XYZ room and you'll be given more details. This invite is specific to you alone. Please do not share it. If you feel someone else also qualifies, let* one *of the core team know for an invite*.

*BATTI on hai for CHANGE!!*”

(Invitation for volunteering session, Core team, November 2017)

### Time Frame Evaluation

The next section presents findings by different phases of the intervention. The analysis led to the emergence of four time frames with each phase indicating important findings that shaped the following one.

#### Phase I: Embedment Phase

The initial embedment phase that occurred from October to December 2017 involved designing the intervention iteratively with quick data analysis every week. This embedment phase also included requesting people to join the page; however, getting this message across till the grassroots was a bit challenging and cumbersome. The group consisted of only 69% of members as some employees did not readily join the page despite having a Facebook account while others created a Facebook account when they were told about the page. This segment was also initiated to examine how the members would react to the new platform that was being designed for the sole purpose of communication. Moreover, it also served the purpose of exploring which posts engaged the employees the most. According to the statistics, 11.8% of the posts were recent updates on the organization's happenings, while 75.7% were “inspirational and thought provoking.” The members mostly used to express themselves through posting photos (51.5%) or links (22.4%), which was the second highest. This period had the highest mean ratings (46.7) and the lowest mean comments (9.2).

The weekly analysis indicated that posts generally revolved around topics like purpose of life, the process of change and transformation, leadership, passion for work, and importance of struggle and success. The comments were based on sharing personal opinions and having intellectual discussions. Even though engagement was progressing, the leaders had to ensure that they had fully gained the employees' trust and certainty. Hence, it was planned to apply the strategy of listening to employees and showing a positive attitude toward them, in the next phases. The leaders intended to target the junior staff, especially the nurses, for the said strategy. The research team decided to first meet the nurses, understand their needs, and then design posts for them.

#### Phase II: Trial Phase

The phase was started with a survey to evaluate the communication strategy. The sample included 32 participants, out of which 12 were ward staff, four trainee physicians, one nursing instructor, eight faculty, and seven staff from intensive care units. Even though the sample size was not enough to generalize the findings, it gave helpful insights to inform the intervention model. The survey results indicated about 80% people used the page to keep themselves updated and stated that Facebook was rated as by far the most successful strategy for engaging employees in the initiative. They specified that they would like the appreciations and updates the most on the Facebook page and would like to see more of such posts along with new knowledge and ideas, discussing real challenges and issues, and following up with suggestions by the leadership. Furthermore, when the employees were asked regarding the changes they could observe since the initiative started, they stated that they could observe more and improved communication between senior doctors and trainee physicians and nurses. They also stated that they could feel positive attitudes around, where individuals were willing to change and were rejuvenated. Through this survey, they further clarified that the reason 75% of them do not comment on posts is because either they did not have the time or they feel they could not relate to the post. These were important insights and informed the strategy for the next phase.

In this phase from January to June 2018, the active users increased to 93%. Analysis of this phase indicated that there were a total of 1,475 posts (16.3% of the total posts) with 49.4 mean ratings, 10.5 mean reactions, and 9.4 mean comments. These statistics suggest that the strategies were effective to a great extent as the employees used the platform to share their achievements and connect with other employees. A further breakdown of this indicates continued energy into the first 3 months with an equal number of average comments of 11 per post. The next 3 months saw a decline in the number of comments per post to 7 per post, while the number of reactions per post slightly increased to 12. This can be related to the fact that the main intervention phase ended in March 2018 and the effects can be seen on the engagement indicators.

The “inspirational and thought provoking' theme had the most posts (52.7%). This was because SOPs were initiated, and infancy stage rudiments started forming during this interval. Informed by the survey, “Person of the Week,” a scheme of “acknowledgment and appreciation” was also introduced at the time, and it had a total of 82 posts. The percentages of post types remained the same with photos (45%) taking the lead followed by statuses (22%).

#### Phase III: Sustainability Phase

Comparatively, the following juncture, i.e., July to December 2018, had lower posts as compared with the previous one and the active members were about the same−91%. The posts were 46.5% of pictures which is the highest; however, this time, the videos had the least posts with only 14.3%. Moreover, the “inspirational and thought provoking” theme was still at the top with 60.7% posts, while “challenges and solutions” had zero posts which was the least. This indicates that the participants were falling out of the challenging circumstances as these were already discussed and resolved in the first two phases. Even though the number of posts remained about the same, the number of mean comments, reactions, and ratings have decreased to 3.8, 6.1, and 23.7, respectively.

An organizational psychologist and Director Patient Experience of Care was appointed to maintain consistency in engagement and also help in making the work environment more conducive. A few training sessions were also organized to teach employees on how to improve their soft skills and also form a strong relationship with mentors and peers. Moreover, the moderators of the group tried to incorporate “informative” posts that focused on the employees' fields. These included posts on organizational psychology, play therapy, teaching methods, research studies, etc.

#### Phase IV: Settlement Phase

Toward the final stage, things started to normalize in the Facebook group and submerged as part of routine. The data deduced for this phase was from January to December 2019, comprising 5,501 posts (60% of the total posts in the study period) with about half of them being photos. The “inspirational and thought provoking” theme posts were still taking a lead with 51.9% followed by “informative” theme with 20.5%. It is interesting to note that all the theme posts had increased in terms of percentages except for “challenges and solutions” which remained zero and “inspirational and thought provoking” decreased by 8.8%. Furthermore, a downward trend can be observed in engagement with respect to mean comments (2.1), mean reactions (5.6), and mean ratings (17.6).

### Top Posts

We also extracted the top 15 posts with the highest ratings, of which seven were from “acknowledgment and appreciation,” five posts from “inspirational and thought provoking,” two from “challenges and solutions,” and one from “recent updates” (Table 6). It was also noted that seven of these highest ratings posts were from the “embedment phase,” which was the highest. On the other hand, four were of the “settlement phase” followed by three belonging to the “sustainability phase” and one from the “trial phase,” which was the lowest.

**Table 6:** Top 15 posts with the highest engagement indicators as indexed by post rating.

**Message**	**Date**	**Theme**	**Type**	**Rating**	**Reactions**	**Comments**
Double Duty is a major Pain point for nursing staff. Though we are working but your input makes a lot of difference. So share your suggestions!!	2018–01	Challenges and solutions	Photo	728	22	228
Congratulations Ms. AA! Person of the week!	2018–09	Acknowledgment and appreciations	Photo	588	153	94
Who can provide more compassionate, empathetic care than a nurse? Who can be more of an advocate for their patients than their nurse? What can we do from tomorrow to make sure that none of our rounds happen without our nurses? None of our plans are made without our nurses' input? Can people describe this picture in just one word? I describe it as Yohsin	2017–12	Inspirational and thought provoking	Photo	512	28	152
Congratulations Dr. BB Person of the Week!	2018–10	Acknowledgment and appreciations	Photo	508	134	80
Congratulations Person of the Week …	2019–07	Acknowledgment and appreciations	Photo	481	122	79
Ok guys here is the question? Q1: What should be the percentage component for art of medicine vs. science of medicine in medical school curriculum especially in Pakistan? Q2: Comment on the 2 pictures below and tell me which will teach both the art and science of medicine more effectively in this age of attention? Read the questions well and answer.	2017–11	Inspirational and thought provoking	Photo	477	12	151
Don't be ashamed of your story … it will inspire others. Who has some real stories to share? Let's get the word out!	2017–12	Inspirational and thought provoking	Photo	455	13	143
So, who can help me with my RPM. I want no patient to ever suffer unnecessarily	2017–11	Challenges and solutions	Status	435	9	139
So proud of you guys! (meeting with chancellor of the university)	2019–04	Acknowledgment and appreciations	Photo	424	134	52
Congratulations to Dr. XX for being awarded the Best innovator award of the year 2018 by the CEO.	2019–03	Acknowledgment and appreciations	Status	417	99	73
Congratulations Person of the Week! SS	2019–12	Acknowledgment and appreciations	Photo	409	92	75
First time ever in history an extremely premature (27 weeks)	2018–9	Recent updates	Photo	392	79	78
Appreciating staff challenge. I appreciated the admission office staff for doing such a tough job. I appreciated Hina our ECHO tech and told her that she is a person with Yohsin (grace)	2017–10	Acknowledgment and appreciations	Status	390	18	118
People who feel called to their careers	2017–12	Inspirational and thought provoking	Link	388	8	124
Hi everyone, I need your help. I am giving a talk tomorrow on “The Art of Pediatrics.” I want to ask all of you a question and need a very personal response from everyone. “Why did you choose Pediatrics?” I want to use some of your responses tomorrow, without using names. Can you please help	2017–12	Inspirational and thought provoking	Status	383	13	119

## Discussion

The present study aimed to evaluate the implementation of an innovative communication strategy employing a social media platform (Facebook group). The findings indicate that the strategy managed to engage the employees with the Facebook group created as indicated by the number of posts (9,085) and consistent number of active members (90%) in 27 months. Multiple employees expressed intent to carry out QI projects voluntarily indicating success of the strategy. The group was created to connect the organization to its employees, understand their needs, inform them how to provide the best employee experience based on their needs, inspire them to lead initiatives for resolution of challenges, and maintain transparency. It can be speculated that when the employees felt that the organization was interested in hearing them to alleviate their pain points, this led to development of a sense of belonging and connection to the organization and a sense of community, facilitating their engagement with the platform. According to Preece and Shneiderman (31), online support communities have a well-defined and narrow community which increases the probability of users to contribute and collaborate. Furthermore, a culture of compassion, i.e., altruism for willingness to collaborate, showing empathy, and a sense of belonging, also fosters greater participation (ibid.) which is critical in the healthcare setting where patient experience hinges upon empathetic care.

The current communication platform proved to be integrative for employee engagement strategies facilitating the four enablers (26). Strategic vision was laid out by leadership through an appropriate communication specifically created for the purpose. An appropriate strategy helps in directing the communication and engaging employees in a productive manner. On top of that, employees receive higher satisfaction when they are informed on how their jobs conform to the organizational mission, the institute's policies and plans, and professional relationships residing in the work environment (Grunig et al., 2002). Another important element for success of the revised communication strategy was the focus on maintaining transparency, keeping leadership approachable, and establishing an informal communication, which in turn promoted integrity. Other similar initiatives based on the social exchange theory (32) have illustrated that when the organizations show concern for the well-being of its employees through practices and systems like transparency, resolving concerns, and consistent and timely feedback, the employees reciprocate through positive work attitudes. The key was to understand employees and engage them in the process of building an integrated work culture based on the new vision. Once the understanding was established, employees sought appreciation; wanted to avail the freedom of voicing and discussing their grievances and be aware of the vision, policies, and their rights; and build relationships. According to Preece and Shneiderman (31), reciprocated emotional support and commitment can highly enhance members' retention as compared to information only. Moreover, it also encourages them to stay engaged with the online community (ibid.).

The findings of this study gave an interesting perspective into engagement of the employees over time. The numbers of posts increased throughout the period, whereas the number of comments and reactions decreased gradually. Apart from this, “challenges and solutions” was the most recurring theme in the first half which at times became overwhelming for mentors to respond to. It was observed that the employees expressed many grievances mostly through comments and reactions as this was the first time they felt that they were important and their opinions and sufferings mattered. However, the numbers decreased over a period of time as efforts were made to find resolutions to the challenges. The number of posts increased overtime indicating acceptance of the platform which filled a major gap. Furthermore, they also felt comfortable to voice their opinions and, more importantly, share their achievements with each other. Likewise, the number of statuses increased after the first 6 months, possibly due to the fact that employees are comfortable to share their opinions and ideas.

The group viewership remained constant and did not decline in any phase. Among the post types, the visual material, i.e., photos, appeared the most on the Facebook page. A possible reason could be because they are considered to be appealing which has also been deduced in other studies, though for other reasons using different platforms (33, 34), and hence, most participants chose to post their content through photos. Additionally, the theme “acknowledgment and appreciation” was viewed the most overtime due to the inauguration of our scheduled post, “Person of the Week.” This post in particular was the most anticipated and received the most likes as it displayed the opportunity to find good in others which promoted selflessness and nourished peer-to-peer relationship. A three-way approach was applied for this scheme which implied that not only a post about the specific person was shared on the group every Friday, but his/her picture was also printed and placed on the notice board of every unit. Every so often, the employees were awarded without informing their supervisors; however, later, it was realized that it is important to keep the supervisors in the loop. Hence, the person also received a copy with appreciation from his/her supervisor. In addition to this, the workers who were awarded with this title were also given the opportunity to meet Pakistani celebrities—Mehwish Hayat and Shehzad Roy—in 2019. This made the employees feel valued possibly promoting organizational citizenship behavior. In short, social media was used to complement other existing modes of communication (35). The most appealing part for the employees was to have the autonomy for nominating a peer—taking away from just leadership having a say. Since all employees were allowed to suggest a nomination, the award was accessible to every individual regardless of their work nature and position in the organization. This led to a reduction in hierarchical gaps of the organizations and further motivated the employees. A remarkable example was when a housekeeping staff was awarded with this title and appreciated by the CEO in his office. This moment was captured and, with their consent (Supplementary Figure 1), was shared to the group and in this study.

Online communities allow more than just letting individuals communicate and share goals. It provides them with the support they seek, through acknowledgments and appreciations, exchange of emotions and grievances, provision of support when needed, and acquaintance on a personal level (36, 37). Human beings possess an inherent need of sharing their emotions and feelings, but if that is suppressed, it results in a disengaged workforce for organizations (38). The study confirms that social media platforms can be used to appreciate employees on a larger scale to make them feel highly motivated. This is very much in line with the key principles of effective positive reinforcement strategies.

The strategy, though implemented before the coronavirus disease 2019 (COVID-19) era, is highly relevant to the workplace context today. The prevalence of the pandemic followed by a lockdown caused struggle among humans in managing emotions and adapting to the changes post COVID-19. Nearly all organizations are also struggling with maintaining professional relationships as they have to put in an extra effort by creating and implementing flexible strategies that cater to employees' needs. In such times, social media platforms have proven to be a strong foundation for maintaining relationships and connections virtually. The Facebook groups are a good source of support in such times. People share their stories, fears, and emotions; discuss how to cope up with the pandemic; and reach out for help when needed. The need has increased more than ever. Hence, organizations must consider adopting those online communication strategies that aim to maintain relationships and monitor the mental well-being of its employees.

Through this initiative, we have realized that the specified Facebook indicators seem promising as a valid measure of emotional engagement. The employees invest extra efforts to add value to their respective organizations once they realize an emotional connection with it. Similarly, the present study indicated that the posts and comments are being done on a voluntary basis; therefore, we believe the driving force for this is their emotional engagement. We further learned that to ensure efficiency and effectiveness, dedicated individuals, clear SOP, and a clear plan around posts are required for this communication strategy. Thus, individuals must possess the knowledge and understanding of the organizational needs as well as human behavior. Moreover, an inclusion of constant feedback is also essential to further sustain effectiveness. The engagement survey can be utilized to assess employees' perceptions about the page and suggestions for improvement.

There are several lessons from the intervention (Table 7). In order to engage the employees with communication strategy, it is important to include content that matters to them, e.g., recognition. Secondly, the employees themselves know better what can motivate and involve them; henceforth, taking their feedback into account at regular intervals is imperative. We found that Cialdini's principle of reciprocity is the most effective for engaging employees, and effort must be made to respond to each employee by the respective leaders and their managers. This requires adequate human resources to not only promptly respond to employees but also respond in a manner that makes them feel heard. This can also be a means of modeling reciprocity which can translate to employees. Another important lesson learned was the need of a clear evaluation plan for the strategy. Qualitative analyses of social media data can reveal critical insights but can also be very time consuming, and resources have to be dedicated accordingly. Apart from this, trainings should be provided to both employees and leaders as not everyone is in the habit of using social media or how to get the best from it. They have to be informed regarding the advantages and must be enabled with the skills required to get started. A phased approach to roll out strategy gradually can be used with the respective supervisors trained to respond to the employees. Moreover, the process of reviewing the data generated from the Facebook group and improvising the strategies themselves was time consuming. Hence, modified strategies with reduced shortcomings that also align with the vision of the organization should be created for communicating through social media. It helps in directing the communication and also how to engage the employees in a productive manner.

**Table 7:** Key lessons learned.

**Domain**	**Lesson learned**
Content	Authentic content should be shared on social platforms.
Leadership role	Increase leaders' involvement by listening to employees and engaging them intellectually for resolving their issues. Leaders should be role models to inspire employees.
Employee engagement	Initially, the engagement level will be high but eventually the participation levels will go down as employees juggle between their work responsibilities and taking part in initiatives.
Trainings	Provide trainings to the employees since everyone is not familiar with using social media in an efficient way. They have to be informed regarding what is in it for them and must be enabled with the skills required to get started. Leaders may require trainings as well.
Principles of persuasion	The principle of reciprocity can be the most effective for engaging employees. Hence, efforts should be made to respond to them.

There were certain strengths and limitations associated with this study. The strength of this study is the utilization of multiple approaches to enhance and measure employee engagement generally and over time, especially in the healthcare organizations residing in LMICs (low- and middle-income countries). It also opens avenues for other organizations to implement a similar strategy in various contexts and situations (e.g., COVID-19). A strong aspect of the implementation was the removal of hierarchical boundaries by encouraging acknowledgment and appreciation of efforts at all levels. Furthermore, the implementation team included an organizational psychologist and an experienced behavior scientist adding rigor to the process. The limitation is that though engagement surveys were planned for evaluating each phase, it was only possible to conduct it once due to lack of human resources. Moreover, the quantitative indicators revealed variation in engagement levels with respect to time frames and themes, but the analysis had failed to incorporate participation on the basis of employee cadre. It was overwhelming to be available to respond to numerous comments by the frontline staff about their pain points which some were newly discovered; hence, a need for teamwork including their supervisors was necessary. Lastly, the analysis was conducted by the research team who were also a part of the workforce; hence, there was a likelihood of bias to emerge in the evaluation.

## Conclusion

Every organization develops its own culture based on the values established due to which the requirements and goals differ. An integrative approach should be applied as a single strategy cannot be efficacious. The current study successfully implemented a social media-based strategy with the aim to engage employees. However, it is important to note that it was not a replacement for other mediums of communication; rather, it complimented the existing strategy. Lastly, along with the appreciation and recognition at multiple levels, it is also important to utilize the strategy to be informed about what matters most to the employees (39).

## Data Availability Statement

The raw data supporting the conclusions of this article will be made available by the authors on reasonable request.

## Ethics Statement

The studies involving human participants were reviewed and approved by Aga Khan University Ethics Review Committee. Written informed consent for participation was not required for this study in accordance with the national legislation and the institutional requirements. Written informed consent was obtained from the individual(s) for the publication of any potentially identifiable images or data included in this article.

## Author Contributions

MR and AH share first authorship. MR conceptualized and designed the paper, contributed toward design of the strategy and led its implementation, led the ongoing analysis, and contributed to manuscript drafts. AH drafted the manuscript and contributed toward interpretation of the findings. SW contributed toward the design of the posts, conducted monthly analysis, and contributed to the draft. HF, SS, and MK prepared the data management and analysis plan and reviewed the analyses and final manuscript. BH provided significant intellectual inputs toward the design and the implementation of the communication strategy and reviewed the final draft. All authors contributed to the article and approved the submitted version.

## Conflict of Interest

The authors declare that the research was conducted in the absence of any commercial or financial relationships that could be construed as a potential conflict of interest.
