# Supplementary figures and images for: Implementation and Evaluation of a Social Media-Based Communication Strategy to Enhance Employee Engagement: Experiences From a Children's Hospital, Pakistan
Source: Front Public Health. 2021 Mar 11;9:584179. doi: 10.3389/fpubh.2021.584179 (PMC7991406; doi:10.3389/fpubh.2021.584179)

Image 1: An employee being recognized by the CEO

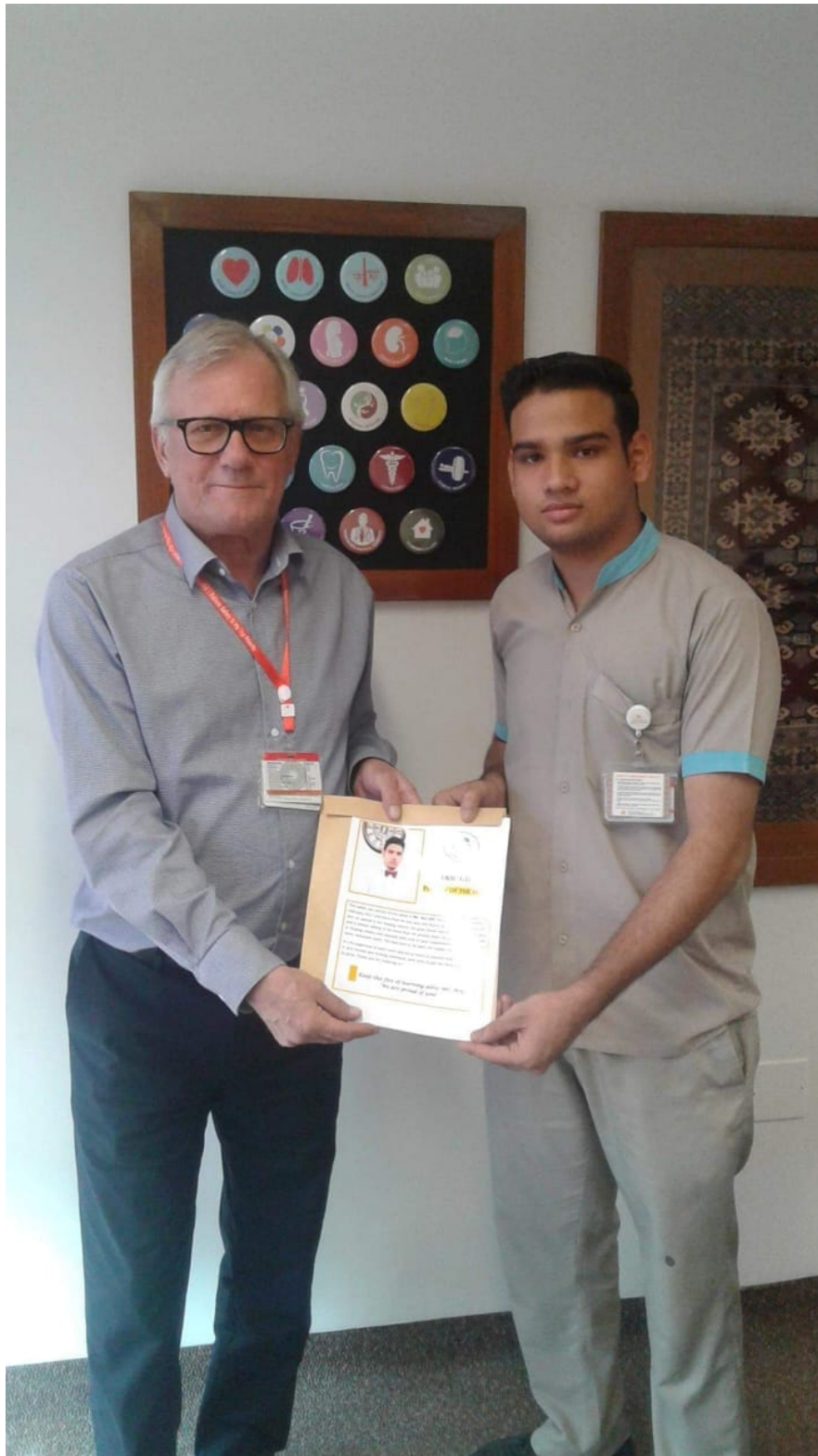

Supplement: Supplementary file 1 [file Data_Sheet_1.PDF]
